# Genome-wide identification and characterization of the growth-regulating factor gene family in oat (*Avena sativa* L.) and functional analysis of *AsGRF17* under drought stress

**DOI:** 10.3389/fpls.2026.1885755

**Published:** 2026-08-12

**Authors:** Jingya Li, Shilun Tong, Kaijun Li, Huiwen Liu, Hang Yin, Mei Yang, Youxu Jiang, Shanshui Zheng, Bing Li, Lulu He

**Affiliations:** College of Animal Science and Technology, Northeast Agricultural University, Harbin, China

**Keywords:** *Avena sativa* L., drought stress, genome-wide, growth-regulating factors, stress response mechanisms

## Abstract

Oat (*Avena sativa* L.) is an important dual-purpose crop used for both food and feed; however, drought stress severely restricts its growth and productivity. Growth-regulating factors (*GRFs*) are key regulators of plant growth and development and are also involved in plant responses to abiotic stresses. Nevertheless, the *GRF* gene family in oat and its potential role in drought tolerance remain largely unclear. In this study, the *GRF* gene family in oat was systematically identified and characterized using bioinformatics analyses, including phylogenetic analysis, conserved motif analysis, and cis-acting element analysis. The expression patterns of oat *GRF* genes under drought, cold, salt, and abscisic acid (ABA) treatments were examined. Among the identified genes, *AsGRF17* was selected for further functional characterization. The *AsGRF17* gene was cloned, and its subcellular localization was analyzed. In addition, transgenic tobacco plants overexpressing *AsGRF17* were generated to evaluate its function in drought tolerance using molecular biology, genetic, and physiological approaches. A total of 21 *GRF* family members were identified in the oat genome. Expression profiling showed that most AsGRF genes were upregulated in response to abiotic stresses, including drought, cold, and salt, as well as ABA treatment, whereas a small number of genes were downregulated. Among these members, *AsGRF17* exhibited the most pronounced transcriptional response in both aboveground and underground tissues. Subcellular localization analysis revealed that *AsGRF17* is localized in the nucleus. Functional characterization further demonstrated that transgenic tobacco plants overexpressing *AsGRF17* displayed significantly enhanced drought tolerance compared with wild-type plants. Under drought stress, the transgenic plants showed increased antioxidant enzyme activities, reduced membrane lipid damage, and elevated soluble sugar content, indicating improved antioxidant capacity and osmotic adjustment ability. These findings suggest that *AsGRF17* positively regulates plant drought tolerance, likely by enhancing antioxidant defense systems and osmotic adjustment capacity. This study provides new insights into the molecular mechanisms underlying *AsGRF17*-mediated drought tolerance in oat and offers a theoretical basis for the genetic improvement and breeding of drought-resistant oat cultivars.

## 1 Introduction

Water constitutes approximately 80%–90% of plant biomass and is indispensable for nearly all physiological and biochemical processes in plants (Brodersen et al., 2019). Drought reduces soil water availability to plants, and is among the most prevalent and complex abiotic stresses affecting plant growth, quality and productivity. Under drought conditions, plants undergo a series of morphological, and physiological changes, water deficit reduces cell turgor and leaf water potential, thereby restricting cell expansion, stomatal aperture, and carbon assimilation. Stomatal closure restricts the diffusion of carbon dioxide to the chloroplasts and constrains water transport and loss, thereby leading to reduced photosynthetic efficiency, lower transpiration rates, and impaired biomass accumulation (Chaves et al., 2009; Farooq et al., 2009). As drought stress intensifies, these effects are further aggravated, leading to pronounced inhibition of plant growth and development (Shao et al., 2009; Osakabe et al., 2014). In addition to constraining gas exchange and photosynthesis, prolonged drought stress disrupts cellular redox homeostasis and promotes the excessive generation of reactive oxygen species (ROS), such as superoxide anion (O_2_^−^), hydrogen peroxide (H_2_O_2_), and hydroxyl radicals (·OH). Overaccumulation of ROS causes oxidative damage to membrane lipids, proteins, nucleic acids, and other cellular constituents, ultimately resulting in metabolic dysfunction, membrane instability, and even programmed cell death (Hasanuzzaman et al., 2020; Pengtao et al., 2024). In agricultural systems, drought reduces soil water availability and is therefore a major constraint on crop growth, yield, and quality (Hussain et al., 2018). Recurrent drought events therefore cause substantial economic losses and pose serious threats to agricultural sustainability, food security, and ecological stability.

To cope with drought stress, plants have evolved sophisticated and coordinated adaptive mechanisms at the physiological and molecular levels (Sato et al., 2024). One of the earliest and best-characterized responses is the activation of the abscisic acid (ABA)-dependent signaling pathway, which promotes stomatal closure and induces the expression of numerous drought-responsive genes (Zhu, 2002; Cutler et al., 2010). In parallel, plants activate antioxidant defense systems composed of enzymatic antioxidants, such as superoxide dismutase (SOD), peroxidase (POD), and catalase (CAT), together with non-enzymatic antioxidants, to detoxify excess ROS and alleviate oxidative injury (Liu et al., 2021). Moreover, plants enhance osmotic adjustment through the accumulation of compatible solutes, including soluble sugars, proline, and soluble proteins, thereby maintaining cellular water status, stabilizing macromolecules, and preserving normal metabolic functions (Zhu, 2002; Basu et al., 2016; He et al., 2020). Therefore, a comprehensive understanding of the physiological and molecular mechanisms underlying plant responses to drought stress is essential for improving crop drought tolerance and ensuring sustainable agricultural production under ongoing climate change.

Growth-regulating factors (GRFs) are plant-specific transcription factors (van der Knaap et al., 2000) that play essential roles in plant growth, development, and responses to environmental stresses (Omidbakhshfard et al., 2015; Kim and Tsukaya, 2015). Members of the GRF family typically contain two highly conserved domains, namely the QLQ and WRC domains (Kim et al., 2003). The QLQ domain shares sequence similarity with the N-terminal region of the yeast SWI2/SNF2 protein that interacts with *SNF11* (Treich et al., 1995), suggesting a role in protein–protein interactions (van der Knaap et al., 2000). The WRC domain contains a nuclear localization signal (NLS) and a DNA-binding motif, enabling *GRFs* to regulate the expression of downstream target genes (Choi et al., 2004). Increasing evidence indicates that *GRF* genes are involved in abiotic stress responses. In kenaf, for example, several members of the *GRF/GIF* module, including *HcGRF3*, *HcGRF21*, and *HcGIF1*, are induced by drought stress. Silencing these genes reduces drought tolerance, whereas their overexpression enhances it, indicating that they function as positive regulators of the drought response (Yue et al., 2025). To date, *GRF* family members have been identified in a wide range of plant species, including 14 in rye (Yang et al., 2023), 14 in maize (Zhang et al., 2008), 22 in soybean (Chen et al., 2019), 27 in alfalfa (Sun et al., 2022), 8 in Astragalus (Li et al., 2023), 9 in *Arabidopsis* (Kim et al., 2003; Kim and Kende, 2004), and 13 in tomato (Khatun et al., 2017). Although *GRF* genes have been implicated in plant stress resistance (Sun et al., 2022), their roles and underlying molecular mechanisms in oat drought tolerance remain largely unclear.

Oat (*Avena sativa L.*) is an annual herbaceous species of the genus *Avena* within the family Poaceae, is an important dual-purpose crop used for both food and forage (Zhang et al., 2025) Owing to its high biomass production, elevated protein content, abundant digestible fiber, and excellent palatability, oat is widely utilized in livestock production. In addition to its forage value, oat is also recognized for its superior nutritional quality. Compared with many other cereal crops, oat grain contains higher levels of β-glucan, unsaturated fatty acids, high-quality protein, dietary fiber, and a range of phenolic compounds. Notably, β-glucan has been shown to play important roles in lowering serum cholesterol, modulating blood glucose, and promoting intestinal health, underscoring the value of oat in functional food development and chronic disease prevention (Whitehead et al., 2014; Wood, 2007). As a result, oat is widely cultivated worldwide as a crop of substantial nutritional and agricultural importance (Mathews et al., 2020). In China, oat is mainly cultivated in arid and semi-arid regions such as Shanxi, Shaanxi, Qinghai, and Gansu (Feng et al., 2020). Compared with many other forage grasses, oat exhibits relatively strong tolerance to several abiotic stresses, including cold, drought, and salinity. However, the increasing frequency and severity of drought caused by climate change have become major constraints on oat growth, yield formation, and quality maintenance. Therefore, uncovering the genetic and molecular basis of drought tolerance in oat is essential for the development of resilient cultivars adapted to water-deficient environments.

Although *GRFs* have been implicated in drought adaptation in several plant species (Hua et al., 2025), their functional roles and regulatory mechanisms in oat remain largely unknown. Therefore, research into the molecular mechanisms of oat drought resistance is crucial for breeding new stress-resistant varieties. In this study, we aimed to systematically characterize the *GRF* gene family in oat and to analyze their expression profiles under drought stress. Among the identified members, we selected the gene (*AsGRF17*) showing the most pronounced transcriptional response to drought as a prime candidate for functional analysis of drought tolerance which we evaluated through phenotypic and physiological assays. Our findings are expected to advance the understanding of *AsGRF*-mediated drought response mechanisms and provide valuable theoretical support and genetic resources for breeding drought-tolerant oat cultivars with stable yield and improved quality under drought conditions.

## 2 Materials and methods

### 2.1 Identification and characterization of oat *AsGRF* genes

The publicly available OT3098 oat genome database was used as the reference genome for the genome-wide identification of the *GRF* gene family, and the OT3098 genome sequence and annotation files were obtained from the Ensembl Plants database (Avena_sativa_OT3098 - Ensembl Genomes 62). 19 GRFs protein sequences from rice, based on the latest annotation release, were retrieved from the Plant Transcription Factor Database (PlantTFDB - Plant Transcription Factor Database @ CBI, PKU) and used as queries for a local blastp search against the oat OT3098 protein database with an E-value threshold of <10^−5^ to identify candidate proteins.

The Hidden Markov Model (HMM) profiles of the conserved GRF domains(InterProScan - InterPro), WRC (PF08879) and QLQ (PF08880), were downloaded from the Pfam database and used to search the oat genome in TBtools-II. Candidate genes identified by both blastp and HMM searches were retained, and sequences containing both conserved domains were considered putative oat GRF genes. These candidates were further verified using the Conserved Domain Database (NCBI-CDD, Home - Conserved Domains - NCBI) to confirm members of the oat *AsGRFs* gene family.

The physicochemical properties of AsGRF proteins, including amino acid length, molecular weight (MW), theoretical isoelectric point (pI), and grand average of hydropathicity (GRAVY), were predicted using ExPASy (https://www.expasy.org/). Subcellular localization was predicted using CELLO(http://cello.life.nctu.edu.tw/).

### 2.2 Chromosomal distribution and phylogenetic analysis of the oat *GRF* gene family

After verifying the consistency of the conserved domains among the candidate genes, chromosomal localization analysis was performed for the 21 identified oat *GRF* genes. Based on their chromosomal distribution and phylogenetic classification, these 21 oat *GRF* genes were renamed, and chromosomal orientations were reversed where necessary. The GRF protein sequences of rice (*Oryza sativa*), wheat (*Triticum aestivum*), barley (*Hordeum vulgare*), and *Arabidopsis thaliana* were retrieved from the Plant Transcription Factor Database (PlantTFDB - Plant Transcription Factor Database @ CBI, PKU). Multiple sequence alignment was conducted using the identified oat GRF protein sequences together with those from these four reference species. Multiple sequence alignment of the 21 identified oat GRF protein sequences and the GRFs sequences from four reference species was performed using the ClustalW algorithm implemented in MEGA11. A phylogenetic tree was subsequently constructed using the Neighbor-Joining (NJ) method with 1,000 bootstrap replicates, while all other parameters were retained at their default settings. The analysis scope is set to All Selected Taxa, the substitution model is Amino acid, the model/method is the default Poisson model, rates among sites are set to Uniform Rates by default, pattern among lineages is set to Same (Homogeneous) by default, gaps/missing data are treated by Pairwise deletion by default, and the number of threads is assumed to be 8. The resulting phylogenetic tree was visualized and annotated using the Interactive Tree of Life (iTOL: Interactive Tree Of Life).

### 2.3 Analysis of gene structure and conserved motifs of the oat *GRF* gene family

To further investigate the functional characteristics and evolutionary relationships of this gene family protein, this study analyzed its conserved domains and motifs. Conserved domains were identified by comparing with the CDD database using the NCBI CD-search tool (Home - Conserved Domains - NCBI, default E-value < 0.01). Motif analysis was performed using the MEME Suite (Introduction - MEME Suite, Version 5.5.9), with full-length protein sequences as input. The core parameters were set as follows: motif discovery mode was set to Classic mode; sequence alphabet was selected as DNA, RNA, or Protein; site distribution was set to Zero or One Occurrence per Sequence; the predicted number of motifs was set to 10; the minimum width of motifs was set to 6 and the maximum width to 50. Finally, TBtools software was used to comprehensively visualize the distribution of gene structures, conserved domains, and motifs. The gene structure, conserved domains, and motif distribution were subsequently visualized using TBtools software.

### 2.4 Collinearity analysis of *AsGRFs* and *cis*-element analysis of *AsGRF* promoter sequences

MCScanX was used to construct interspecific collinearity maps, and the collinearity relationships within and between species were visualized using the Dual Synteny Plot module in TBtools. Promoter sequences of *AsGRF* genes were obtained from the oat genome by extracting the 2,000 bp region upstream of the translation initiation codon (ATG). Cis-acting elements in the promoter regions of *GRF* genes were predicted using the PlantCARE database (PlantCARE, a database of plant promoters and their cis-acting regulatory elements), and the results were visualized with the Simple BioSequence Viewer in TBtools.

### 2.5 Plant materials and treatments

Due to its favorable agronomic traits, the oat cultivar ‘Qingyin No. 1’ (*Avena sativa* L.) was selected as the experimental material for the expression pattern analysis in this study. Seeds with uniform fullness were selected and sown in small black square pots (6.2 cm in diameter, 5.5 cm in height, and 4.8 cm in basal width) and irrigated with half-strength Hoagland nutrient solution. All plants were grown in an artificial climate chamber under a 16 h light/8 h dark photoperiod, at a light intensity of 8000 Lx, with day/night temperatures of 25°C/20°C and relative humidity maintained at 55%/70%. After 3 weeks of growth, seedlings with uniform growth status were subjected to the following treatments: low temperature at 4°C, 15% PEG6000 (to simulate drought stress) (Jia et al., 2021), 150 mmol/L NaCl (salt stress), 150 mmol/L NaHCO_3_ (alkaline stress), and 100 μmol/L abscisic acid (ABA) (Yang et al., 2025). The low-temperature treatment was conducted in a programmable incubator using a gradient cooling regime, beginning at 24°C and decreasing to 4°C at a rate of 2°C per hour. Drought, salt, and alkaline stress treatments were applied by irrigating the soil with the corresponding solutions prepared in half-strength Hoagland nutrient solution. ABA treatment was administered by foliar spraying, with the application endpoint defined as the point at which droplets just began to roll off the leaf surface, thereby ensuring uniform treatment. Root and shoot samples of oat seedlings were collected at 0 h (control), 3 h, 6 h, 12 h, 24 h, and 48 h after treatment for analysis of *AsGRFs* gene expression patterns. In addition, at the booting stage, root, stem, leaf, flower, leaf sheath, and panicle tissues were collected for tissue-specific expression analysis. All samples were collected with three biological replicates, immediately frozen in liquid nitrogen, and stored at −80°C until further use.

### 2.6 Real-time quantitative polymerase chain reaction analysis of *AsGRFs* gene expression

The expression patterns of *AsGRF* genes in different tissues, as well as their responses to abiotic stress and ABA treatment, were analyzed by quantitative real-time PCR (qRT-PCR). Total RNA was first extracted from oat samples using an Ultrapure RNA Extraction Kit (CWBIO, China). First-strand cDNA was then synthesized according to the manufacturer’s instructions using HiScript^®^ II Q RT SuperMix for qPCR (+gDNA wiper) (Vazyme, China). Gene-specific primers for *AsGRF*s were designed using the Integrated DNA Technologies (Integrated DNA Technologies | IDT) online platform (**Supplementary Table 1**), and *AsUBC* (**Supplementary Table 1**) was used as the internal reference for PCR amplification and electrophoretic validation. qRT-PCR was performed using ChamQ Universal SYBR^®^ qPCR Master Mix (Vazyme, China) on a Quantagene Q225 Real-Time PCR system (Novogene, China). Each sample included three independent biological replicates and three technical replicates. The qRT-PCR reaction system was set up in a total volume of 10 μL, containing 5 μL of ChamQ Universal SYBR^®^ qPCR Master Mix, 0.4 μL of each forward and reverse primer, 0.5 μL of diluted oat cDNA, and 3.7 μL of RNase-free water. The amplification was performed using a two-step protocol: an initial pre-denaturation at 95°C for 30 s, followed by 40 cycles of denaturation at 95°C for 10 s, and annealing/extension at 60°C for 30 s. Relative expression levels of *AsGRFs* gene were calculated using the comparative cycle threshold method (2^−ΔΔCt^) (Livak and Schmittgen, 2001).

### 2.7 Isolation and cloning of the *AsGRF17* gene

Degenerate primers (*AsGRF17*-F/*AsGRF17*-R; **Supplementary Table 1**) designed using Primer 5.0 software (Applied Biosystems) were used to amplify the coding sequence (CDS) of *AsGRF17* with 2×Phanta^®^ Max Master Mix (Vazyme, China). The 50 μL PCR reaction mixture for *AsGRF17* gene cloning consisted of 25 μL of 2× Phanta^®^ Max Master Mix, 2 μL each of forward and reverse primers, 2 μL of undiluted oat cDNA, and 19 μL of RNase-free water. The PCR amplification program was performed as follows: initial denaturation at 95°C for 3 min; 35 cycles of denaturation at 95°C for 15 s, annealing at 61°C for 15 s, and extension at 72°C for 30 s; and a final extension at 72°C for 5 min.The amplified product was ligated into the pCE2TA/Blunt-Zero vector (Vazyme, China), transformed into *Escherichia coli DH5α* competent cells, and subsequently sent to Rui’s Biotechnology Co., Ltd. (Harbin, China) for sequencing. After sequence verification, the obtained *AsGRF17* CDS was submitted to the NCBI database under GenBank accession number PX841308.

### 2.8 Subcellular localization of AsGRF17 protein

The linear *AsGRF17* fragment was amplified by PCR using the specific primers PC1301S-*AsGRF17*-F/R (**Supplementary Table 1**) containing homologous arms. The PC1301S expression vector was linearized by KpnI digestion and purified, followed by homologous recombination with the linear *AsGRF17* fragment to generate the *AsGRF17*-1301S construct. Rice seeds were dehulled, surface-sterilized with 75% ethanol and 40% sodium hypochlorite, and germinated on 1/2 MS medium for 8–10 days. Seedlings were collected, and roots and leaves were removed. The remaining tissues (mainly pseudo-stem) were cut into 0.5 mm strips, transferred to a 50 mL conical flask, and incubated in 0.6 M mannitol for 15 min in the dark. After filtration through a 200-mesh sieve, the filtrate was discarded and fresh enzyme solution was added. Samples were then digested in the dark at 28°C with shaking at 80 rpm for 4–6 h. The digestion mixture was filtered again to remove the enzyme solution, and the residue was washed with W5 solution by gentle shaking for 10 s, followed by filtration. The protoplast suspension was transferred to a new centrifuge tube, and this washing step was repeated 2–3 times until a total volume of approximately 45 mL was collected. The suspension was centrifuged at 1200 rpm for 3 min at room temperature, the supernatant was discarded, and the pellet was resuspended in W5 solution and centrifuged again; this step was repeated twice. Finally, the protoplasts were resuspended in an appropriate volume of W5 solution (0.8–1 mL, depending on protoplast yield) and examined under a microscope (Chen et al., 2006). For transient transformation, 1.5–10 μg of *AsGRF17*-1301S plasmid and 100 μL of protoplast suspension were sequentially added to a 2.0 mL round-bottom centrifuge tube and gently mixed. Subsequently, 120 μL of PEG4000 solution was added and mixed gently. After incubation at room temperature for 20 min, 480 μL of W5 solution was added to terminate the transformation. The protoplasts were collected by centrifugation at 1500 rpm for 3 min at room temperature, and the supernatant was discarded. The pellet was resuspended in 1 mL of W5 solution and incubated in the dark at 28°C overnight (12–48 h). After centrifugation at 1500 rpm for 1 min, the supernatant was removed, leaving approximately 100 μL to resuspend the protoplasts. GFP fluorescence was then observed using a laser confocal microscope (FV10,Olympus,Japan).

### 2.9 Plant transformation and the generation of transgenic plants

The pBWA(V)BS-*AsGRF17*-Linker-OsGFP fusion expression vector was introduced into *Agrobacterium tumefaciens* strain GV3101 and subsequently used to transform wild-type (WT) tobacco (*Nicotiana tabacum* cv. K326), generating *AsGRF17*-overexpressing plants (*AsGRF17*-OE). Genomic DNA was first extracted from each putative transgenic line, and PCR amplification of the selectable marker gene Bar was amplified by PCR using *barGRF*-F/R (**Supplementary Table 1**) to performed for preliminary screening. Positive lines were initially identified by agarose gel electrophoresis, and T1-generation seeds were harvested.

The T1 seeds were germinated on MS medium containing glufosinate, and well-developed T1 seedlings were selected and transplanted. Subsequently, total RNA was extracted from T1 seedlings of each line and reverse-transcribed into cDNA. Using the obtained cDNA as the template, *NtActin* as the internal reference gene, and untransformed tobacco as the WT control, the relative expression levels of *AsGRF17* were determined by qRT-PCR, and positive T2-generation seeds were obtained. Based on the expression levels, three lines with relatively high *AsGRF17* expression were selected, and their T2-generation seeds were further screened with glufosinate. The expression levels of *AsGRF17* were then re-evaluated, and T3-generation seeds were obtained. In subsequent experiments, T3-generation seeds were used to investigate the regulatory effects of *AsGRF17* on plant responses to drought stress.

### 2.10 Measurement of physiological indicators and gene expression levels in T3 generation tobacco lines under drought stress

T3-generation tobacco seeds were sown in small black square pots (6.2 cm in diameter, 5.5 cm in height, and 4.8 cm at the base). Plants were supplied with half-strength Hoagland nutrient solution throughout cultivation. The growth chamber was maintained under a 16 h light/8 h dark photoperiod, at a light intensity of 8000 Lx, with day/night temperatures of 25°C/20°C and relative humidity of 55%/70%.After 3 weeks of growth, WT, OE#1, OE#6, and OE#9 tobacco lines were treated with 15% PEG6000, and phenotypic changes were recorded. Tobacco samples were collected on the 2nd and 5th days of continuous treatment with PEG6000, respectively. All samples were prepared in triplicate and immediately stored at −80°C until further analysis. Peroxidase (POD) activity was determined by the guaiacol method (Polle et al., 1994), malondialdehyde (MDA) content was measured using the thiobarbituric acid (TBA) method (Puckette et al., 2007), and superoxide dismutase (SOD) activity was assayed based on the xanthine–xanthine oxidase reaction (McCord and Fridovich, 1969). Soluble sugar content was determined using the anthrone colorimetric method (Morris, 1948), and hydrogen peroxide (H_2_O_2_) content was measured using the titanium sulfate method (Brennan and Frenkel, 1977). Physiological parameters were measured in triplicate technical replicates for each biological replicate. All physiological indices were analyzed using commercial assay kits provided by Beijing BoxBio Science & Technology Co., Ltd. (Beijing, China).

### 2.11 Data analysis

All experiments in this study were independently repeated three times using the same procedures, and the data are presented as the mean ± standard error of the mean (SEM). Data processing was performed using Excel 2024, and statistical analyses, including Student’s t-test, one-way analysis of variance (ANOVA), and *post-hoc* comparison, were conducted using IBM SPSS Statistics 27. Figures were prepared using PowerPoint 2024, MEGA 11.0.13, Origin 2021, and TBtools-II.

## 3 Results

### 3.1 Identification and characterization of AsGRFs in oats

**Figure 1:**
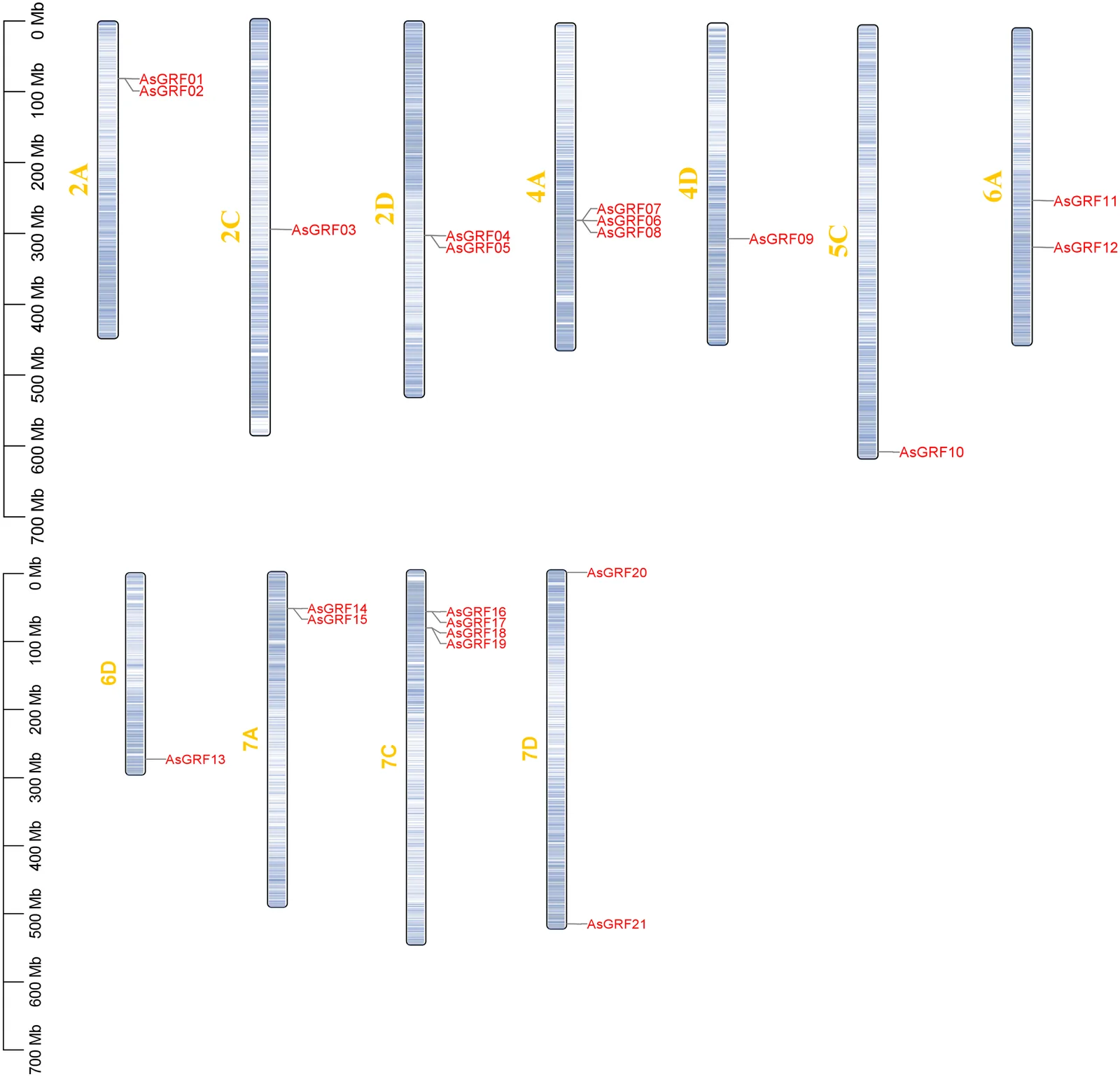
Chromosomal mapping of the 21 *GRFs* of oat.

To identify *GRF* family members in the oat genome, the whole-genome dataset was screened using HMM profiles corresponding to the WRC (PF08879) and QLQ (PF08880) domains, together with Blastp-based sequence searches. Subsequent sequence alignment and conserved domain verification led to the identification of 21 putative *GRF* genes in oat. Based on phylogenetic relationships and chromosomal positions, these genes were designated as *AsGRFs*. Chromosomal mapping revealed that the 21 *AsGRFs* genes were unevenly distributed across chromosomes 2, 4, 6, and 7, with the highest number located on chromosome 7C, whereas chromosomes 2C, 4D, and 6D each harbored only one gene (**Figure 1**). Physicochemical characterization showed that the encoded AsGRFs proteins varied in length from 168 to 593 amino acids, with predicted molecular masses ranging from 18.38 to 63.34 kDa, theoretical isoelectric points from 4.84 to 9.55, instability indices from 46.98 to 75.33, and GRAVY values from -1.073 to -0.133 (**Supplementary Table 2**). These properties suggest that oat GRF proteins are predominantly hydrophilic and may be structurally unstable. Subcellular localization prediction using CELLO2.5 indicated that all AsGRFs proteins are localized in the nucleus (**Supplementary Table 2**), supporting their putative role as nuclear transcription factors involved in gene regulation.

### 3.2 Phylogenetic and protein sequence analysis of the *AsGRFs* gene family

**Figure 2:**
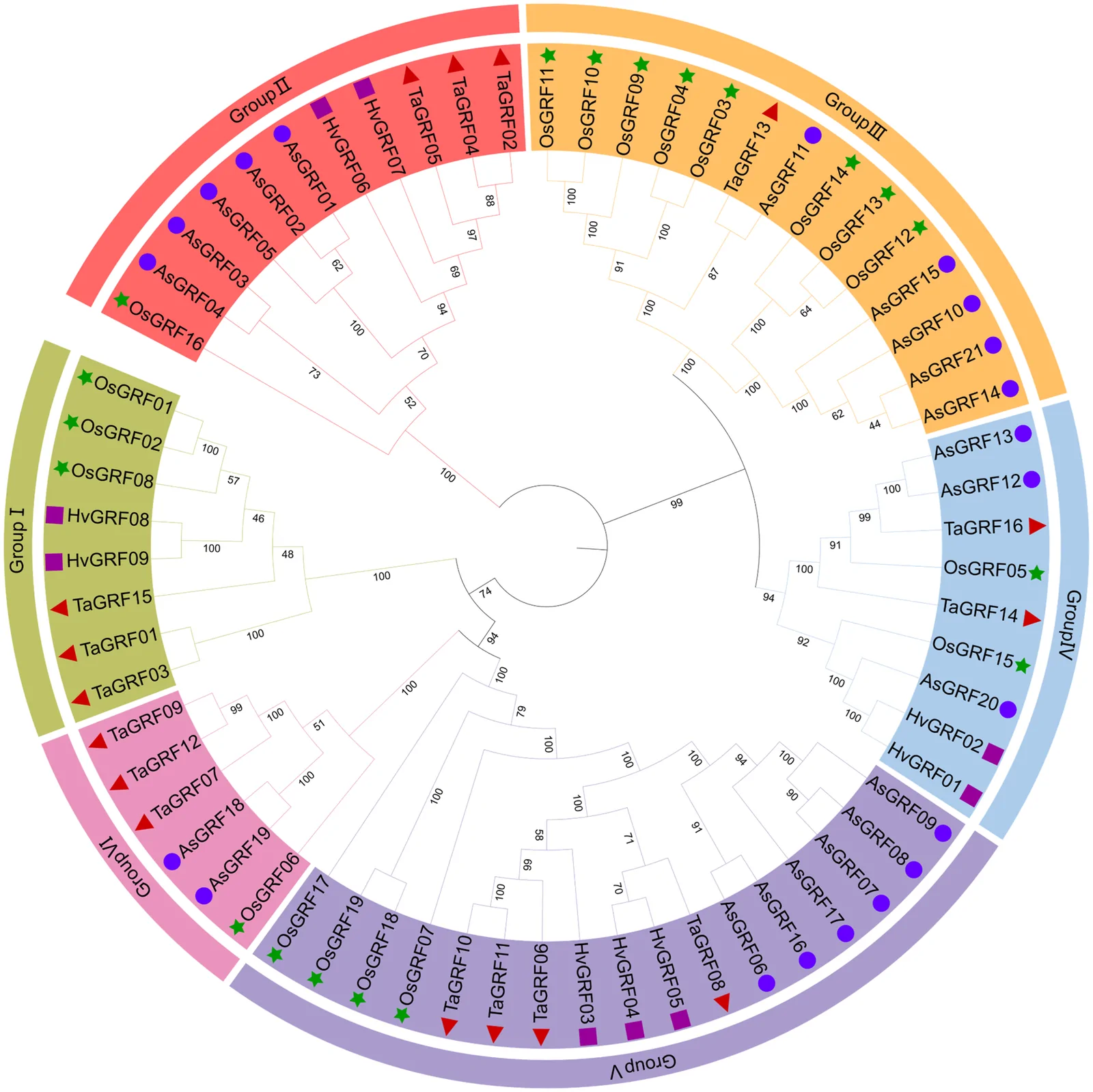
Phylogenetic analysis of *GRF* genes in oat, rice, barley and wheat. The amino acid sequences of GRF proteins from oat, rice, wheat and barley were used for the phylogenetic analysis. The phylogenetic tree was constructed with MEGA 11.0 using the maximum likelihood method with 1000 bootstrap replicates. Different symbols on the phylogenetic tree represent different species.

**Figure 3:**
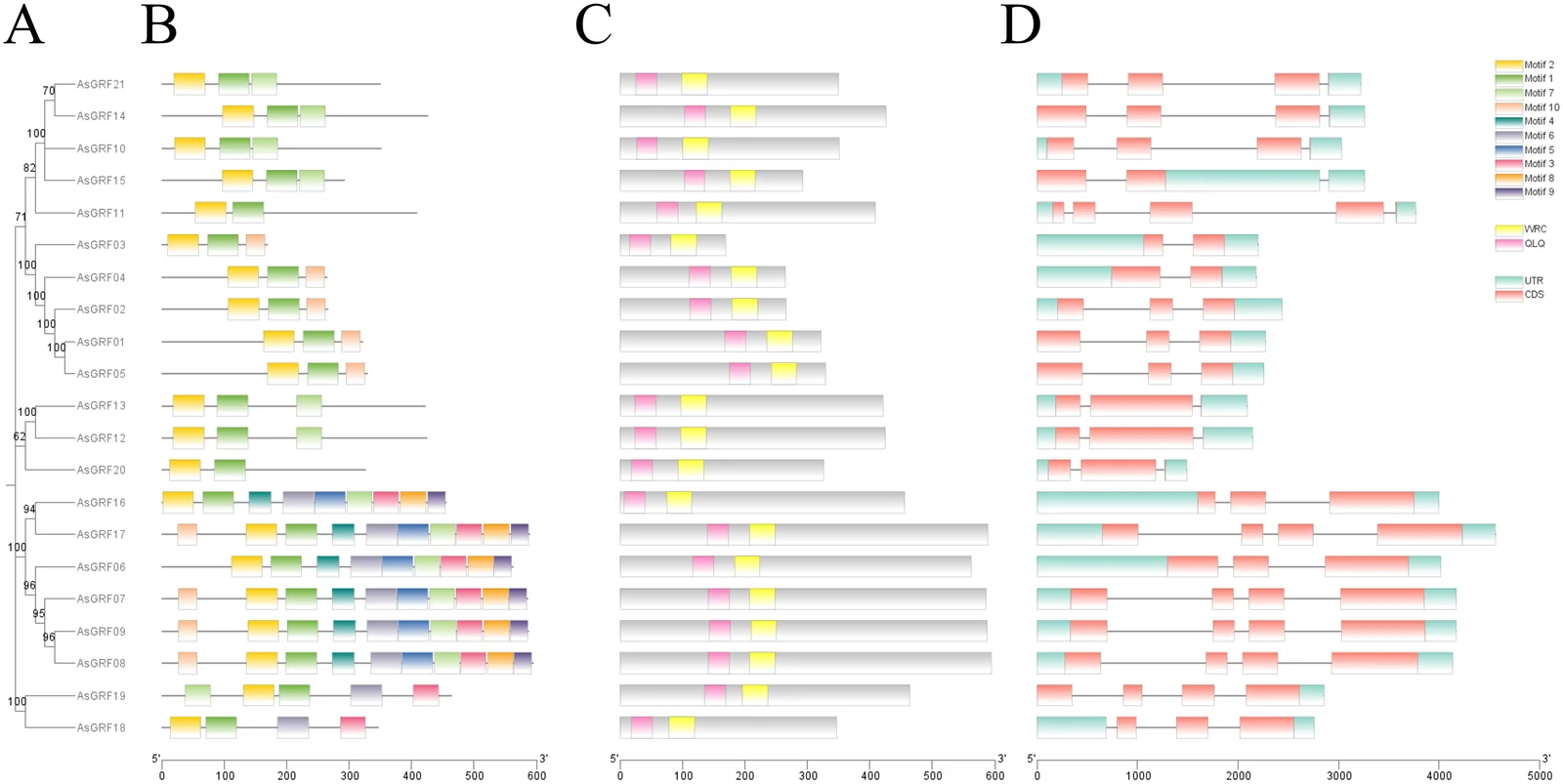
Sequence structure analysis of AsGRFs in oat. **(A)** Phylogenetic analysis of the amino acid sequences of 21 AsGRFs using MEGA 11.0. **(B)** Motif distribution of AsGRF proteins. Different motifs (1–10) are indicated by different colors. The sequence logos for each motif are provided in **Supplementary Figure 1**. **(C)** Domain distribution of AsGRF proteins. Different domains are indicated by different colors. **(D)** Exon–intron and CDS structures of AsGRFs.

To elucidate the evolutionary relationships among members of the oat *GRF* gene family, a phylogenetic tree was constructed using 65 GRF protein sequences from oat, rice, barley, and wheat. Phylogenetic analysis indicated that the GRF family could be classified into six major subfamilies (Groups I–VI), with oat GRFs distributed across five subfamilies, from Group II to Group VI (**Figure 2**). To further investigate the evolutionary diversification of the 21 AsGRFs proteins, conserved motif analysis was performed, and a total of 10 motifs were identified. The sequence information for each motif is provided in **Supplementary Figure 1** and **Supplementary Table 3**. Each AsGRF protein contained 3 to 10 motifs, with some motifs shared among all family members and others restricted to specific subgroups (**Figures 3A, B**). For instance, Motifs 1 and 2 were present in all AsGRFs proteins, conserved motifs 3 and 6 were shared by AsGRF06, AsGRF07, AsGRF08, AsGRF09, AsGRF16, AsGRF17, AsGRF18, and AsGRF19, all of which were grouped into Subfamilies V and VI. Motifs 4, 5, 8, and 9 were specifically present in AsGRF06, AsGRF07, AsGRF08, AsGRF09, AsGRF16 and AsGRF17, corresponding exclusively to members of Subfamily V. Notably, motif 10 was detected in AsGRF06, AsGRF07, AsGRF08, AsGRF09, and AsGRF17 from Subfamily V, as well as in AsGRF01, AsGRF02, AsGRF03, AsGRF04, and AsGRF05 from Subfamily II. The subfamily-specific or preferential distribution of these conserved motifs suggests that AsGRF proteins within the same subfamily possess relatively conserved motif architectures, which is consistent with their phylogenetic relationships.

CDD analysis further confirmed that all 21 AsGRFs proteins contained the conserved WRC and QLQ domains (**Figure 3C**). Motif 1 corresponded to the WRC domain and Motif 2 to the QLQ domain, both of which were highly conserved. Closely related genes on the phylogenetic tree exhibited similar conserved motif architectures; for example, AsGRF06, AsGRF07, AsGRF08, AsGRF09, AsGRF16,and AsGRF17 clustered closely and displayed highly similar motif compositions. Exon–intron structure analysis revealed that *AsGRFs* genes from different phylogenetic groups exhibited distinct gene structures, whereas genes within the same group generally shared similar structural features (**Figure 3D**). The conservation of gene structure among members of the same subfamily was consistent with their phylogenetic relationships. Specifically, oat *GRF* genes contained 2 to 4 introns, with three introns being the most common pattern.

### 3.3 Collinearity and gene duplication analysis of *AsGRFs*

**Figure 4:**
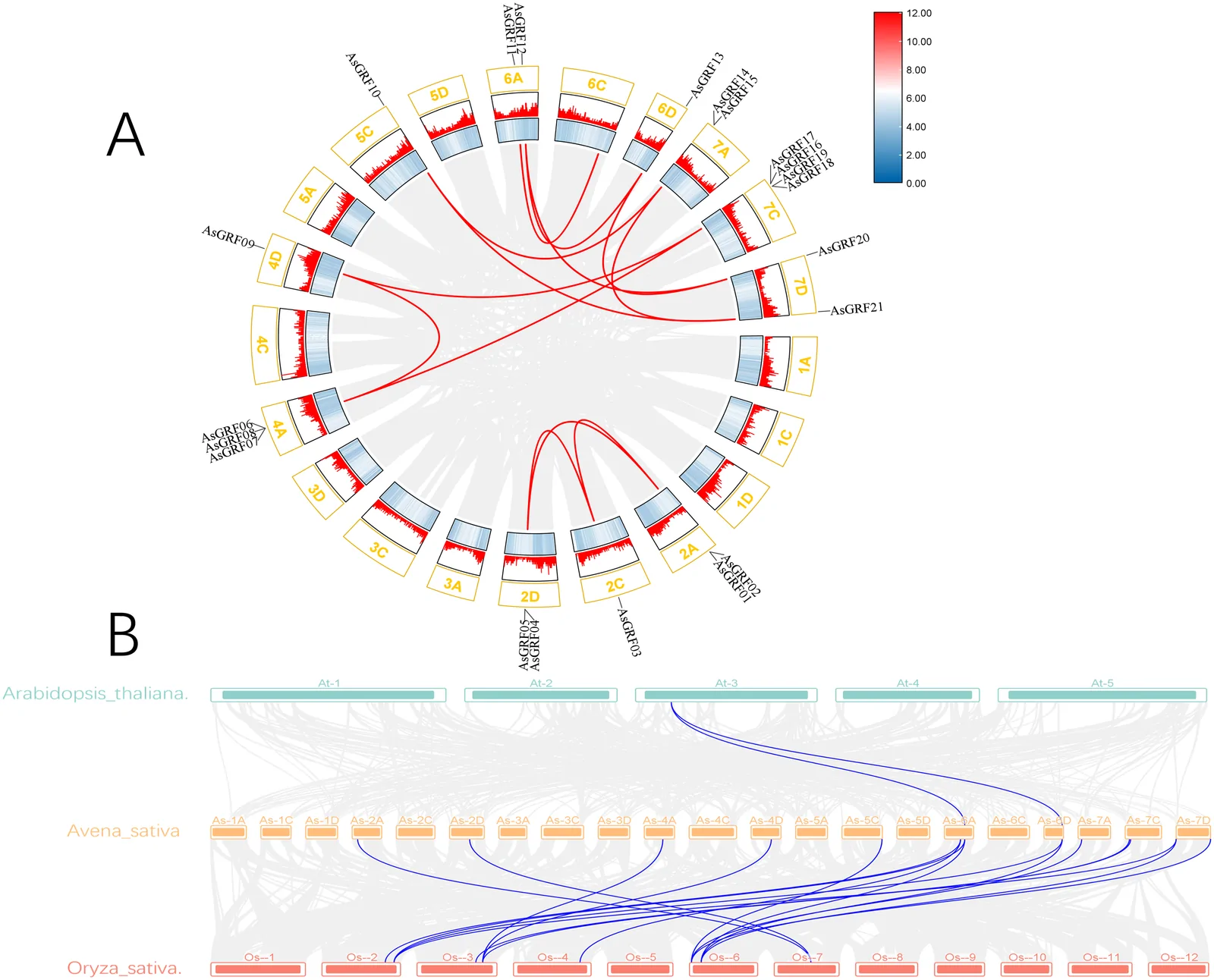
Gene collinearity analysis of the *AsGRFs* in oat. **(A)** Collinearity analysis within the oat species. The 21 *AsGRFs* were mapped to 11 chromosomes. The grey lines indicate all synteny blocks in the oat genome, and the duplicated gene pairs of *AsGRFs* are connected with red lines. **(B)** Collinearity analysis between species. Homology analysis of *GRF* gene families among *Avena sativa*, *Arabidopsis thaliana* and *Oryza sativa*. The red color represents syntenic *GRFs*, and the gray lines show the collinear blocks of the plant genome.

To gain a deeper understanding of the functions and evolutionary mechanisms of the *AsGRFs* gene family in oat, the intraspecific and interspecific collinearity of *AsGRFs* family members were analyzed using the MCScan toolkit in TBtools software. Based on the intraspecific collinearity analysis, the 21 *AsGRFs* genes were distributed across different chromosomes, and 13 gene pairs were identified as segmentally duplicated genes (**Figure 4A**). To further investigate the selective pressure acting on *AsGRFs* gene pairs in oat, the Ka/Ks ratios were calculated (**Supplementary Table 4**). The Ka values of the gene pairs ranged from 0.001374 to 0.275356, whereas the Ks values ranged from 0.012904 to 0.393195. Notably, all gene pairs exhibited Ka/Ks values between 0.062042 and 0.700304, which were all below 1. These results indicate that the duplicated *AsGRFs* genes have undergone strong purifying selection during evolution, thereby reducing amino acid substitutions caused by nonsynonymous mutations and preserving protein structure and function.

To further elucidate the evolutionary relationships of *GRF* genes across species, syntenic blocks involving *GRF* genes among oat, rice, and *Arabidopsis* were mapped. Between oat and *Arabidopsis*, two pairs of syntenic genes were identified, with the corresponding genes located on chromosome 3 of *Arabidopsis* and chromosomes 6A and 6D of oat. In contrast, 17 pairs of syntenic genes were detected between oat and rice, among which chromosome 6A of oat contained the highest number of syntenic pairs (four pairs), followed by chromosome 7D with three pairs (**Figure 4B**). Overall, the syntenic relationship between oat and rice was markedly stronger than that between oat and *Arabidopsis*.

### 3.4 Analysis of cis-acting elements of *AsGRFs*

**Figure 5:**
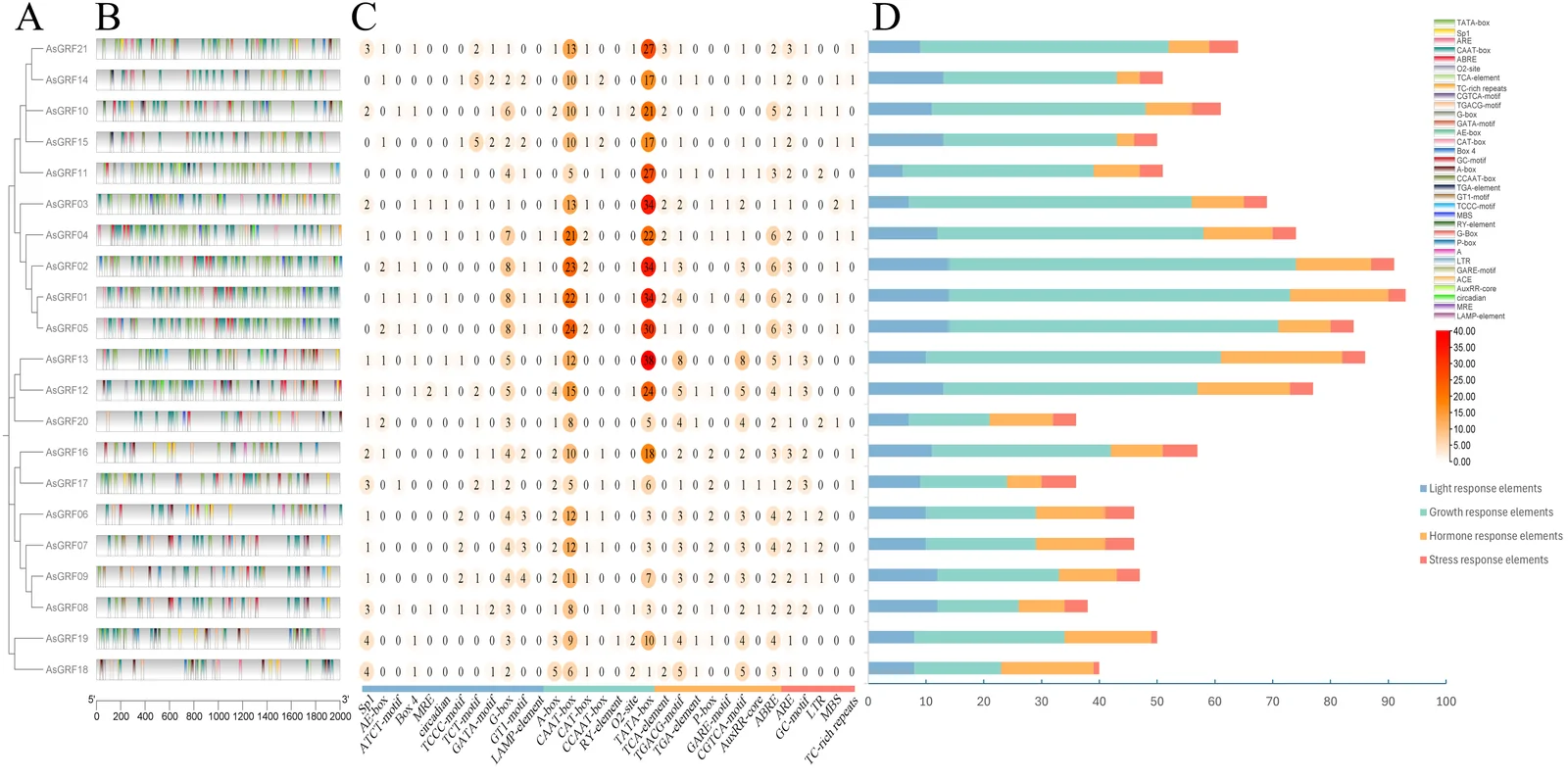
Cis-elements in putative promoter regions of *AsGRFs* in oat. **(A)** Phylogenetic analysis of the amino acid sequences of 21 AsGRFs using MEGA 11.0. **(B)** Distribution of predicted cis-acting regulatory elements within the 2000-bp upstream promoter regions of each *AsGRFs* gene, with different colors representing distinct types of cis-acting elements. **(C)** Heatmap showing the abundance of cis-acting regulatory elements in the promoter regions of *AsGRFs* genes; the numbers indicate the copy number of each element, and the color gradient from light to dark represents increasing element abundance. **(D)** Functional classification and statistical summary of the identified cis-acting regulatory elements.

To investigate the potential regulatory mechanisms underlying *AsGRFs* gene expression, the 2.0-kb promoter sequences upstream of the ATG start codon of each *AsGRFs* gene were analyzed for cis-regulatory elements. A total of 32 major cis-acting elements were identified and grouped into four functional categories, including light-responsive, growth-responsive, hormone-responsive, and stress-responsive elements. Subsequently, the number of cis-acting elements belonging to each category was quantified for individual *AsGRFs* genes (**Figures 5C, D**). The 12 light-responsive elements comprised SP1, AE-box, ATCT-motif, Box4, MRE, circadian-related elements, TCCC-motif, TCT-motif, GATA-motif, G-box, GT1-box, and LAMP-element. Seven growth-responsive elements were detected in the promoter regions of *AsGRFs* genes, including the meristem expression-related element (CAT-box), metabolic regulation-related element (O2-site), seed-specific regulation element (RY-element), CCAAT-box, A-box, CAAT-box, and TATA-box. In addition, eight hormone-responsive elements were identified, including the abscisic acid-responsive element (ABRE), auxin-responsive elements (TGA-element, AuxRR-core), gibberellin-responsive elements (P-box, TARE-motif), jasmonic acid-responsive elements (CGTCA-motif, TGACG-motif), and the salicylic acid-responsive element (TCA-element). Meanwhile, the stress-responsive elements included the low-temperature-responsive element (LTR), defense- and stress-responsive element (TC-rich repeats), drought-inducible element (MBS), antioxidant-responsive element (ARE), and elements associated with hypoxia and wounding stress responses (GC-motif) (**Figures 5A, B**). Collectively, these results suggest that *AsGRFs* may function not only in plant developmental regulation but also in responses to abiotic stress, light signaling, and hormone-mediated regulation, with different members of the same subfamily potentially exhibiting distinct response patterns.

### 3.5 *AsGRFs* respond to abiotic stress in the aerial parts and roots

To further characterize the expression profiles of *AsGRFs* genes, oat plants were used as experimental material, and qRT-PCR was performed to examine their transcriptional responses under different abiotic stress conditions and abscisic acid (ABA) treatment. Based on the phylogenetic classification, two representative genes were selected from each of the five subfamilies containing *AsGRFs* members. In addition, because *AsGRF11* and *AsGRF20* formed relatively distant branches within their respective subfamilies, these two genes were also included, resulting in a total of 12 *AsGRFs* genes for expression analysis.

**Figure 6:**
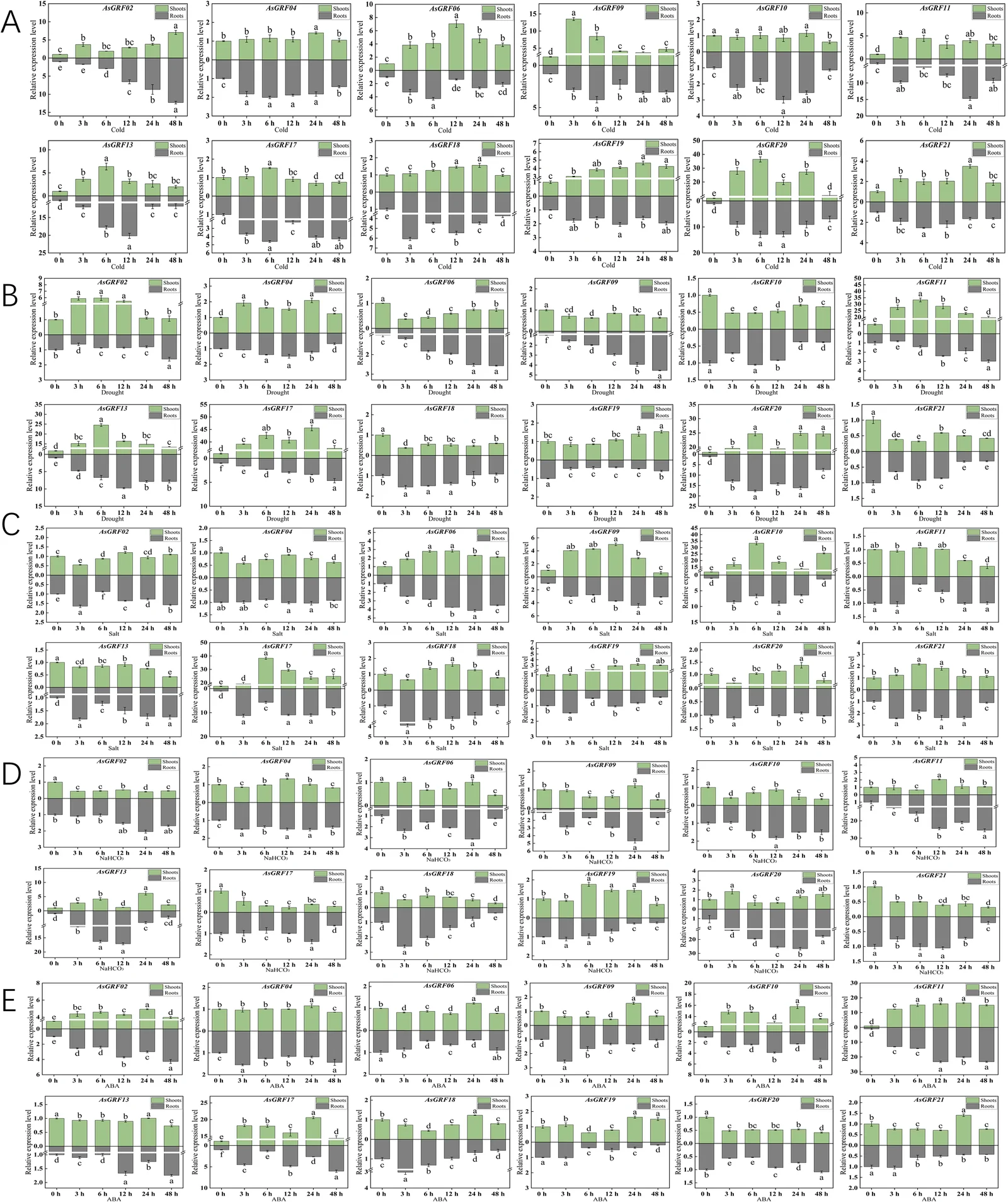
Analysis of expression patterns under abiotic stresses. **(A–E)** show the expression patterns of genes under cold, drought, alkali stresses and ABA treatment, respectively. Using ANOVA, the letters denote statistically significant differences (a=0.05).

The results showed that these genes displayed pronounced time-dependent expression patterns in response to the different treatments. Under low-temperature stress (**Figure 6A**), the expression levels of all 12 *AsGRFs* genes in both aboveground and belowground tissues were significantly higher at all examined time points (3, 6, 12, 24, and 48 h) than those in the 0 h control, indicating that low-temperature stress induces *AsGRFs* gene expression. Under drought stress conditions (**Figure 6B**), only *AsGRF10* and *AsGRF21* exhibited a decreasing expression trend in both aboveground and belowground tissues. In addition, the expression levels of *AsGRF06*, *AsGRF09* and *AsGRF18* in aboveground tissues, as well as that of *AsGRF19* in belowground tissues, were significantly reduced at 3 h after treatment. Under non-saline stress conditions (**Figure 6C**), only six genes *AsGRF06*, *AsGRF09*, *AsGRF10*, *AsGRF17*, *AsGRF18* and *AsGRF21* showed consistently upregulated expression in both aboveground and belowground tissues. Moreover, among the 12 selected genes, the number of genes exhibiting unchanged or increased expression levels in belowground tissues under saline stress was greater than that observed in aboveground tissues. Under alkaline stress conditions (**Figure 6D**), with the exception of *AsGRF13*, *AsGRF19* and *AsGRF20*, which exhibited significantly increased expression in aboveground tissues following stress treatment, all other genes showed varying degrees of downregulation. In belowground tissues, except for *AsGRF19*, whose expression progressively decreased with increasing stress duration, the expression levels of all other genes displayed an initial increase followed by a subsequent decrease. Under ABA treatment (**Figure 6E**), the expression levels of all genes except *AsGRF20* showed an increasing trend in aboveground tissues, reaching a peak at 24 h and subsequently declining to varying degrees by 48 h. In belowground tissues *AsGRF06*, *AsGRF19* and *AsGRF21* exhibited a progressive decrease in expression with increasing treatment duration, whereas *AsGRF09* and *AsGRF18* reached their highest expression levels at 3 h and then continuously declined over time. In contrast, the remaining genes showed varying degrees of upregulation, characterized by a fluctuating expression pattern.

Overall, *AsGRFs* responded dynamically to abiotic stresses and ABA treatment. Among them, *AsGRF17* showed the most pronounced and consistent induction under drought, low temperature, salt stress, and ABA treatment in both aboveground and underground tissues, particularly under drought stress. Under drought conditions, *AsGRF17* exhibited the highest fold change and the most marked upregulation trend in aboveground tissues, while its expression in underground tissues increased progressively with prolonged drought exposure. Therefore, *AsGRF17* was selected for further analysis.

### 3.6 Separation of *AsGRF17* and its characteristics

**Figure 7:**
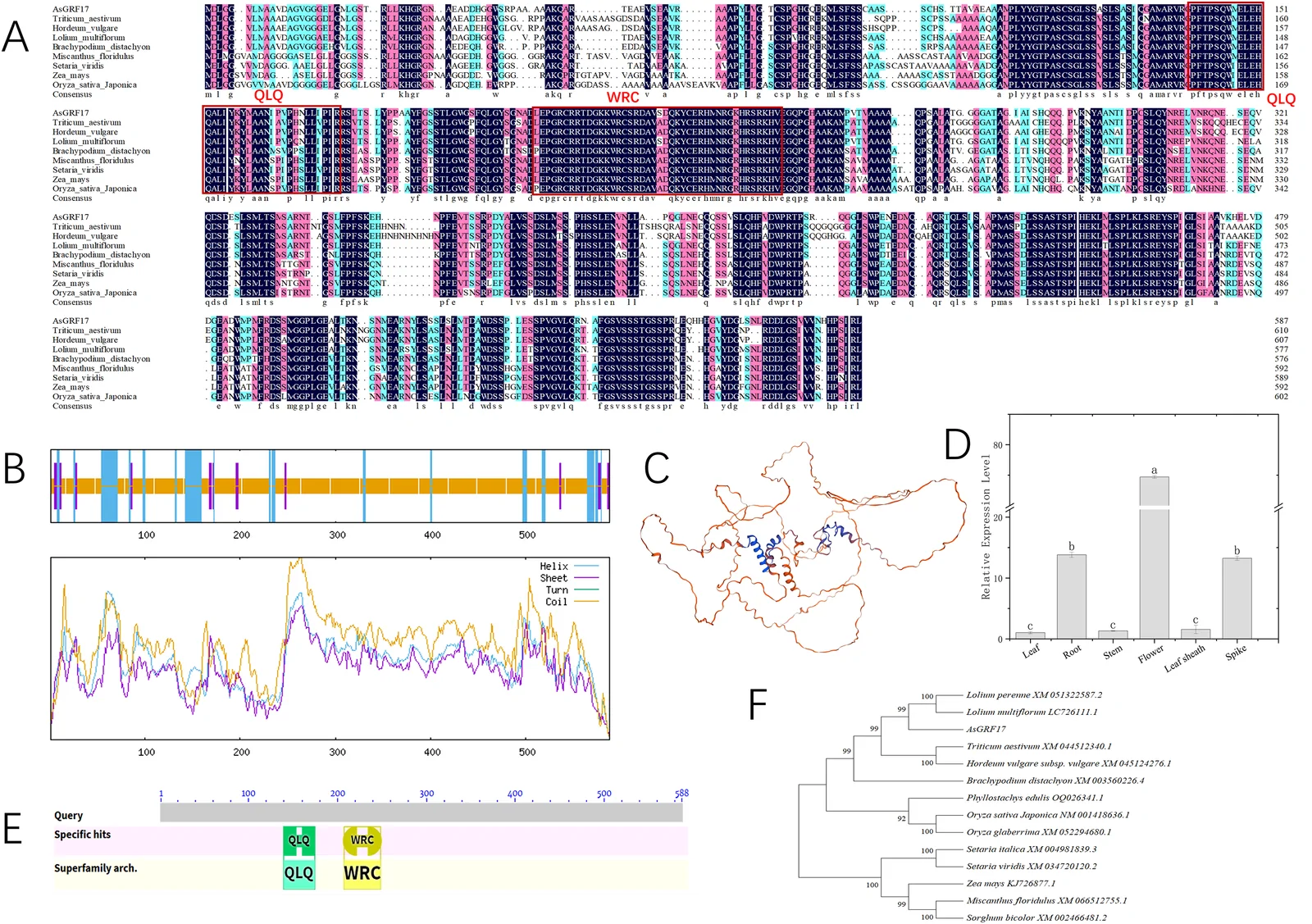
Amino acid sequence analysis of *AsGRF17* and expression pattern of coding genes. **(A)** Amino acid sequence alignment. **(B)** Composition of the protein structure of the *AsGRF17* gene. **(C)** 3D protein model of *AsGRF17*. **(D)** Expression level of the *AsGRF17* gene in oat at different tissue sites. **(E)** Domains of the *AsGRF17* protein. **(F)** Phylogenetic tree of *AsGRF17*. Using ANOVA, the letters denote statistically significant differences (a=0.05).

Based on the above findings, *AsGRF17* was cloned from oat leaves to further investigate its function. The full-length CDS of *AsGRF17* is 1,767 bp and encodes a protein of 588 amino acids containing the conserved QLQ and WRC domains (**Figures 7A, E**). Protein structure analysis revealed that *AsGRF17* is composed of 13.78% α-helices, 4.42% extended strands, and 81.80% random coils, with the latter representing the predominant structural element (**Figure 7B, C**). Phylogenetic analysis showed that *AsGRF17* clustered in the same branch as the corresponding genes from annual ryegrass (*Lolilum multiflorum*) and perennial ryegrass (*Lolium perenne)*, suggesting a close evolutionary relationship and potential functional similarity (**Figure 7F**). Tissue-specific expression analysis indicated that *AsGRF17* was predominantly expressed in roots, flowers, and spikes (**Figure 7D**).

### 3.7 Subcellular localization of AsGRF17

**Figure 8:**
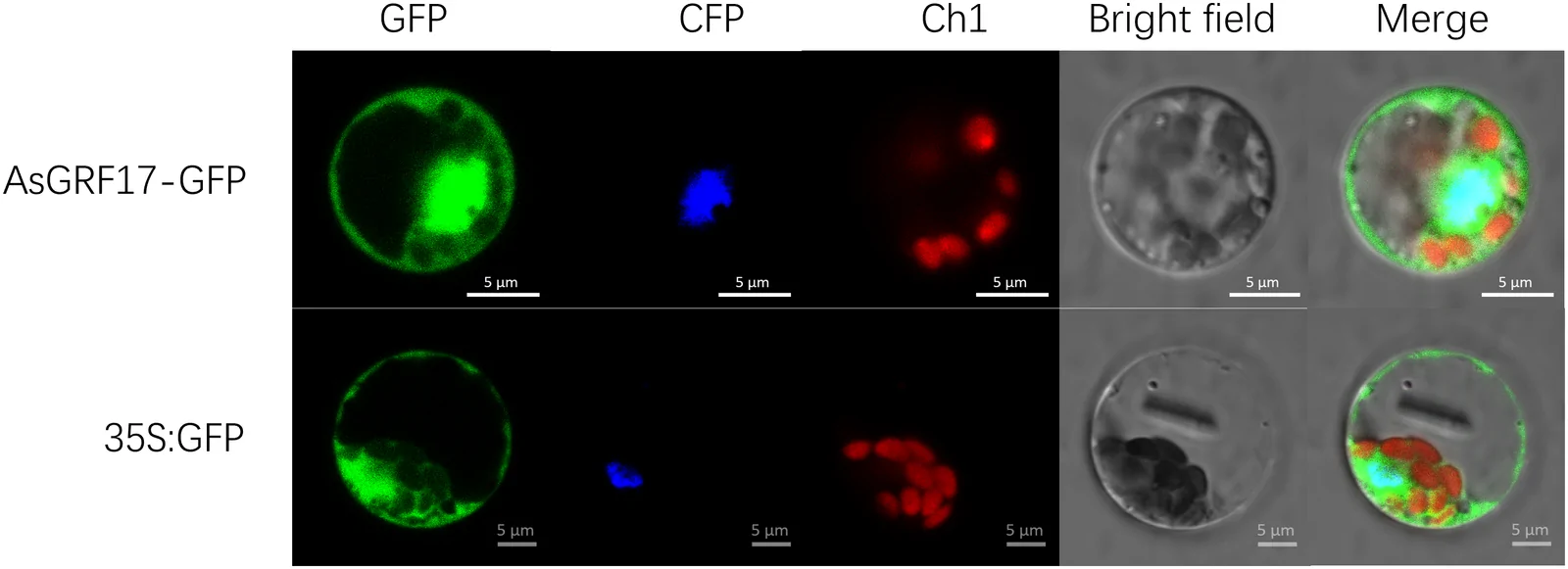
Subcellular localization of AsGRF17.

To validate the predicted subcellular localization of *AsGRF17*, the constructed *AsGRF17*-1301S expression vector was co-transformed with the nuclear marker GHD7-CFP into rice protoplasts. Confocal laser scanning microscopy revealed overlap between the *AsGRF17*-1301S fluorescence signal and the nuclear marker, indicating that the *AsGRF17* protein is localized in the nucleus, in agreement with the prediction results. The control in this experiment was Agrobacterium transformed with the empty 1301 vector containing the GFP tag. In addition, fluorescence signals were also detected in the cytoplasm, suggesting that *AsGRF17* may also localize to the cytoplasm (**Figure 8**).

### 3.8 Role of *AsGRF17* in drought tolerance

**Figure 9:**
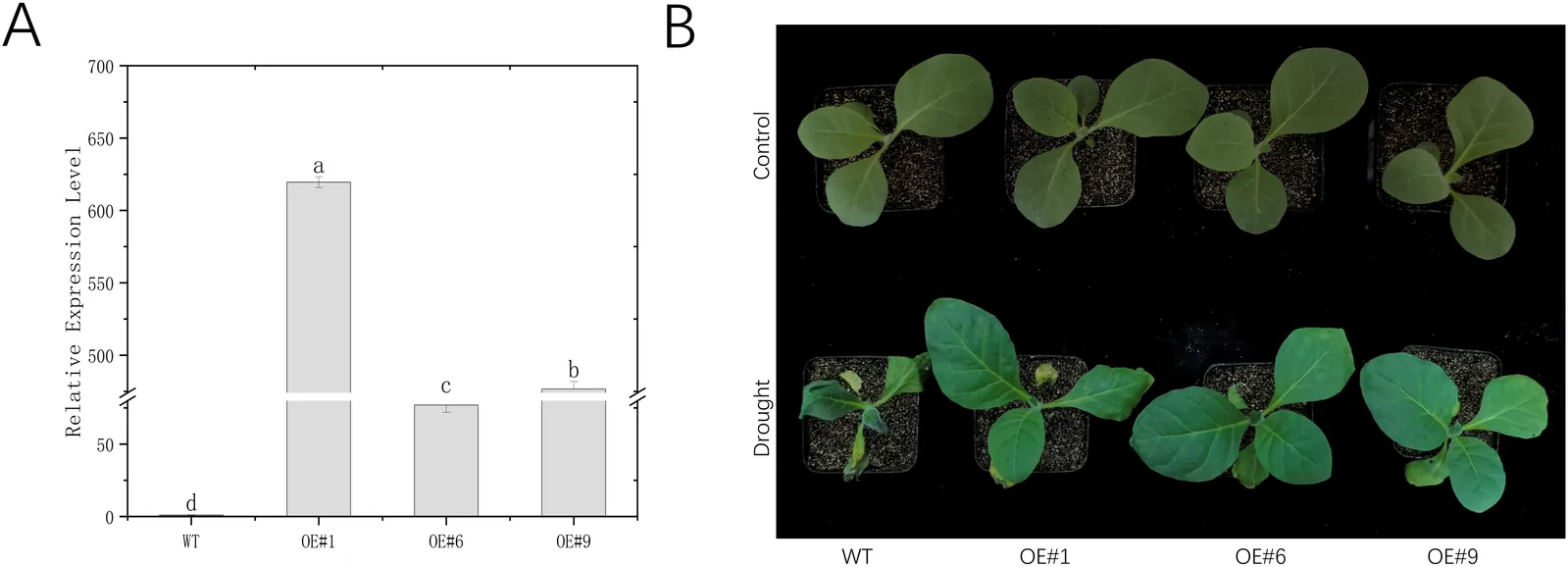
Identification and phenotype of *AsGRF*-transformed lines. **(A)** *AsGRF17* relative expression levels in the transgenic tobacco lines. **(B)** Phenotypes of transgenic tobacco plants overexpressing *AsGRF17* under control condition and drought stress. Using ANOVA, the letters denote statistically significant differences (a=0.05).

To investigate the role of *AsGRF17* in drought stress response, overexpression lines were generated and verified by PCR detection of the bar marker gene (**Supplementary Figure 2**). qRT-PCR analysis confirmed that *AsGRF17* transcript abundance in the OE#1, OE#6, and OE#9 lines was 619.5-, 76.9-, and 476.6-fold higher than that in the wild type, respectively (**Figure 9A**). Under normal growth conditions, the transgenic lines were phenotypically indistinguishable from the wild type. Following 5d of 15% concentration PEG6000-induced osmotic stress, both transgenic and wild-type plants displayed leaf yellowing and wilting; however, these symptoms were markedly more severe in the wild type (**Figure 9B**). These results suggest that *AsGRF17* overexpression confers enhanced drought tolerance in transgenic plants.

### 3.9 Research on the physiological progress of transgenic plants overexpressing *AsGRF17* under drought stress

**Figure 10:**
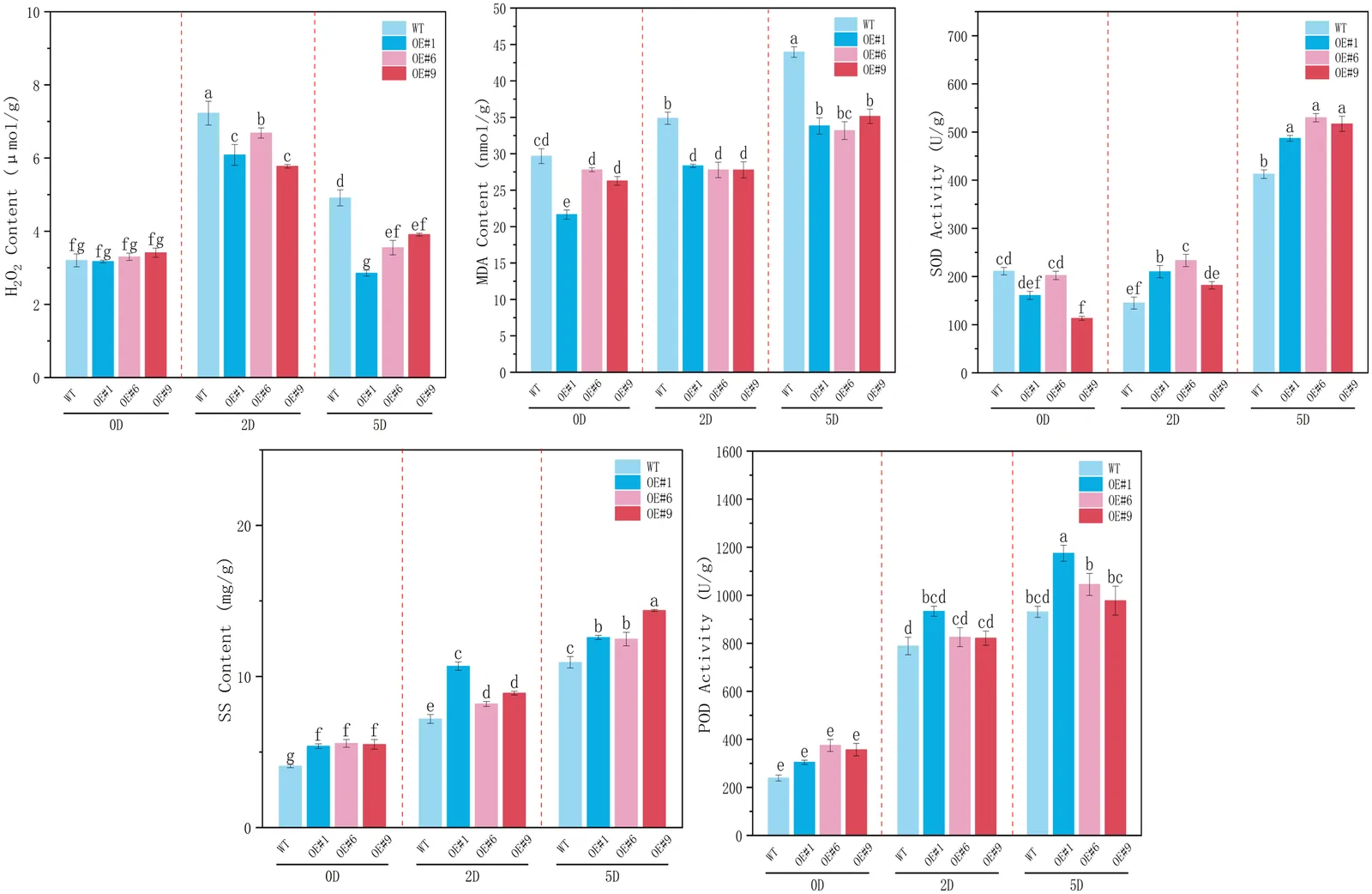
Modifications in the physiology of transgenic tobacco plants overexpressing *AsGRF17* under drought stress. Using ANOVA, the letters denote statistically significant differences (a=0.05). H2O2, hydrogen peroxide. MDA, malondialdehyde. SOD, superoxide dismutase. POD, peroxidase. SS, soluble sugar.

To further elucidate the role of *AsGRF17* in stress resistance, physiological responses of transgenic tobacco plants under drought stress were assessed. Compared with wild-type (WT) plants, POD and SOD activities in transgenic plants increased progressively with stress duration and peaked on day 5 of drought treatment, with significantly higher levels observed in the overexpression lines than in the WT plants. H_2_O_2_ content in all lines initially increased and then declined as stress progressed, reaching a maximum on day 2; notably, H_2_O_2_ levels were significantly higher in WT plants than in transgenic plants. In addition, MDA content in transgenic plants was significantly lower than that in WT plants under both normal and drought conditions. Soluble sugar content in transgenic tobacco plants was also significantly higher than that in WT plants under control conditions and after 5 d of drought treatment (**Figure 10**).

## 4 Discussion

The *GRF* gene family has been widely identified and functionally characterized in diverse plant species. Previous studies have shown that *GRF* genes participate in multiple aspects of plant growth and development, including floral organ development (Liu et al., 2014), leaf and cotyledon growth (Kim et al., 2003), root development (Hewezi et al., 2012), and seed size regulation (Li et al., 2016). In kenaf, functional analyses demonstrated that silencing *HcGRF3* and *HcGRF21* reduced drought and salt tolerance, whereas their overexpression enhanced drought tolerance (Yue et al., 2025), indicating that specific GRF members may act as positive regulators of abiotic stress tolerance. In *Arabidopsis*, overexpression of miR396 suppresses the expression of six *GRF* genes and also represses *GIF1*, a transcriptional co-activator that interacts with *GRFs*, thereby impairing cell proliferation and producing a narrow-leaf phenotype (Liu et al., 2009). These findings suggest that reduced *GRF* activity can restrict cell proliferation and organ growth, whereas sustained or enhanced *GRF* activity may contribute to maintaining plant growth under stress conditions. The regulatory relationship between miR396 and *GRF* genes further supports this view. In rice, blocking miRNA-mediated repression can directly induce the expression of *OsGRF6*, thereby promoting tillering and spikelet development and ultimately increasing grain yield (Gao et al., 2015). Similarly, under UV-B irradiation, stress-induced upregulation of miR396 leads to downregulation of *GRF* genes, resulting in inhibited cell proliferation and reduced leaf growth (Casadevall et al., 2013). These observations indicate that *GRF* genes generally function as positive regulators of growth-related processes, particularly cell proliferation and organ expansion. Therefore, maintaining relatively high *GRF* expression or activity under drought stress may help alleviate drought-induced growth inhibition. In addition to their roles in development, increasing evidence indicates that *GRF* genes are involved in plant responses to abiotic and biotic stresses. In rice, *OsGRF6* has been reported to enhance grain yield and resistance to bacterial leaf blight (Yuan et al., 2024), while *OsGRF7* improves plant salt tolerance (Chen et al., 2024). In wheat, *TaGRF3-2A* has been implicated in growth regulation (Huang et al., 2021), and *GRF*-related genetic manipulation has been used to compensate for the negative effects of dwarfing alleles in spring triticale (Divashuk et al., 2021). In maize, the *ZmMPK3–ZmGRF1* module promotes plant growth under salt stress by enhancing cell proliferation (Yin et al., 2026). Collectively, these studies demonstrate that *GRF* genes are not only key regulators of plant growth and development but also participate in responses to diverse environmental stresses.

In this study, we identified the oat *GRFs* gene family and characterized its bioinformatic features and expression patterns under different stress conditions. A total of 21 *AsGRFs* genes were identified, which differs slightly from the numbers reported in rice (Choi et al., 2004), barley (Wang et al., 2023), and *Arabidopsis* (Kim et al., 2003; Kim and Kende, 2004). This variation likely reflects species-specific differences in gene family size and highlights the evolutionary diversification and adaptability of *GRF* genes (Cao et al., 2016). A previous study on the oat cultivar *Avena _ sang* identified 28 *GRFs* members in the *Avena_sang* database (Xu et al., 2026), whereas only 21 members were identified in the OT3098 database used in the present study. Comparative analysis of the 21 *AsGRF* family members identified from the OT3098 oat genome with the 28 *AsGRF* genes annotated in the *Avena sang* oat genome showed that only 10 OT3098 *AsGRF* genes shared 100% sequence identity with their corresponding *Avena sang* homologs across the full-length sequence. This discrepancy is mainly attributable to differences in database resources and update status. Since the *GRFs* gene family was first identified in 2003, it has been reported in multiple species and functionally validated in diverse biological processes. However, subfamily classification based on phylogenetic analysis has varied among studies. For example, a 2026 study in upland cotton classified the *GRFs* family into four subfamilies (Feng et al., 2026; Kim et al., 2003), a 2023 study in ryegrass divided it into six subfamilies (Yang et al., 2023), and a 2024 study in sweet cherry classified it into five subfamilies (Deng et al., 2024). Because oat and ryegrass both belong to the Poaceae family, and phylogenetic analysis indicates a closer evolutionary relationship between oats and ryegrass (**Figure 7F**), we classified the oat *GRFs* family into six subfamilies based on the clustering of the 21 *AsGRFs* genes, following the classification approach used in ryegrass. The 21 oat genes were distributed across five of these six subfamilies (**Figure 2**). The rice, wheat, and barley sequences used for phylogenetic reconstruction in this study were obtained from the PlantTFDB transcription factor database. Notably, PlantTFDB currently annotates 19 rice *GRFs* genes, which differs from the 12 genes reported in earlier literature. This difference reflects database expansion and continuous updates since the initial identification of the *GRF* family in rice in 2004 (Choi et al., 2004).

Physicochemical analysis showed that most AsGRFs proteins are basic and hydrophilic. All 21 members had instability indices greater than 40, indicating that they are likely unstable proteins and may be prone to degradation or denaturation (**Supplementary Table 2**). The conserved QLQ and WRC domains are key features underlying the evolutionary conservation of GRFs proteins (Meng et al., 2022). Consistent with this, members within the same subfamily displayed similar sequence lengths, physicochemical properties, and motif compositions, whereas these characteristics differed among subfamilies. Previous studies have shown that the *GRF* family responds to multiple hormonal signals (Zhang et al., 2018). In the present study, numerous hormone-responsive cis-elements, including ABRE, P-box/GARE-motif, and TCA-element, were identified in the promoter regions of *AsGRFs* genes, indicating that these genes are likely regulated by multiple hormones. In addition, several stress-responsive elements, such as MBS and LTR, were detected, supporting the involvement of the *GRF* family in stress responses (**Figure 5**).

Based on the number of *AsGRFs* gene in each subfamily, two to three genes were selected from each subfamily for expression pattern analysis. To maximize representation, the selected genes were chosen from different branches within each subfamily whenever possible. The results showed that most genes were upregulated in both aboveground and belowground tissues under abiotic stress and ABA treatment, whereas only a few genes were downregulated in either aboveground or belowground tissues (**Figure 6**). These findings suggest that the *GRF* family participates broadly in abiotic stress responses.

Tissue-specific expression analysis further showed that *GRFs* genes were highly expressed in growing and developing tissues but weakly expressed in mature stems and leaves, consistent with previous reports (Cheng et al., 2023) (**Figure 7D**). qRT-PCR analysis indicated that *AsGRF17* was most strongly expressed in flowers. Since *GRFs* genes have been implicated in flowering time regulation and floral organ development in rice (Liu et al., 2014), this pattern suggests a potential role for *AsGRF17* in reproductive development. In addition, the relatively high expression of *AsGRF17* in roots implies a positive role in root growth, consistent with previous findings in tomato showing that GRF overexpression promotes root development (Hua et al., 2025). Subcellular localization analysis confirmed that *AsGRF17* is localized in the nucleus (**Figure 8**), consistent with prediction results. Together, these analyses provide a basis for future studies on the roles of the GRF family in flowering regulation, floral organ development, and root growth.

Plants possess sophisticated antioxidant defense systems that protect cells from oxidative damage and maintain metabolic homeostasis by scavenging stress-induced reactive oxygen species (ROS), thereby improving tolerance to environmental stress. Previous studies have suggested that, besides regulating growth and development, the *GRF* family may also play important roles in stress adaptation by regulating the expression of genes involved in stress responses and metabolic homeostasis (Omidbakhshfard et al., 2015). The results of this study indicate that, in terms of phenotypic changes, overexpression of the *AsGRF17* gene significantly enhanced the tolerance of transgenic tobacco to drought stress (**Figure 9B**), and associated physiological indicators showed changes (**Figure 10**). In this study, 5 d after drought treatment, H_2_O_2_ levels in *AsGRF17*-overexpressing tobacco plants were significantly lower than those in the wild type, whereas SOD and POD activities were significantly higher (**Figure 10**). As major protective enzymes, SOD and POD act synergistically to remove excess ROS, reduce membrane lipid peroxidation, and maintain normal membrane function, thereby enhancing plant adaptation to stress (Jin et al., 2010). SOD catalyzes the conversion of O_2_^−^ into H_2_O_2_ and O_2_, while POD further converts H_2_O_2_ into H_2_O. MDA is a widely used indicator of membrane damage under abiotic stress (Ayala et al., 2014). In this study, MDA levels increased in all plants after 5 d of drought treatment; however, the increase was significantly smaller in *AsGRF17*-overexpressing lines than in the wild type, indicating that *AsGRF17* overexpression alleviates drought-induced membrane injury. Similar findings were reported in tomato, where overexpression of *SiGRF4* reduced MDA and H_2_O_2_ levels and enhanced antioxidant enzyme activity under drought stress, thereby improving drought tolerance (Hua et al., 2025). In addition to antioxidant defense, osmotic adjustment is another key mechanism for drought adaptation. The accumulation of osmoprotectants such as proline, soluble proteins, and soluble sugars is a common response to drought stress and helps maintain cellular structure and function under osmotic constraints (Blum, 2017), thereby sustaining plant performance under dehydration (Blum, 2017). After 5 d of drought exposure, soluble sugar content increased significantly in all plants, and this increase was more pronounced in *AsGRF17*-transgenic tobacco than in the wild type (**Figure 10**). Collectively, these results suggest that *AsGRF17* enhances drought tolerance by promoting osmotic adjustment, maintaining membrane integrity, and modulating antioxidant enzyme activity.

Numerous studies have investigated the *GRF* gene family and its associated regulatory networks. Kim et al. first demonstrated that AtGIF1/AN3 acts as a transcriptional co-activator that interacts with GRF transcription factors (Kim and Kende, 2004). AtGIF1/AN3 does not directly bind DNA; instead, it regulates cell proliferation-related developmental processes, particularly leaf growth and organ formation, through its association with GRF proteins. This discovery established the basis for the concept of the GRF–GIF transcriptional regulatory complex. Subsequent studies further revealed that AN3/GIF1 is able to recruit SWI/SNF chromatin-remodeling complexes, such as BRAHMA/BRM, to GRF-associated target regions, thereby influencing chromatin structure and transcriptional regulation. The SWI/SNF–AN3/GIF regulatory module has been shown to be essential for proper leaf development (Vercruyssen et al., 2014). Based on these findings, the enhanced drought tolerance observed in *AsGRF17*-overexpressing plants in the present study may not be attributable solely to the expression change of *AsGRF17* itself. Rather, it may involve the coordinated function of *AsGRF17* with *GIF* co-regulators and related transcriptional regulatory machinery, thereby enhancing plant adaptability to drought stress.

Under drought stress, ABA-dependent signaling pathways play pivotal roles in plant drought responses by promoting osmotic adjustment and enhancing antioxidant defense Capaci (Cutler et al., 2010; Vishwakarma et al., 2017). Drought-induced ABA accumulation activates downstream stress-responsive networks through the *PYR/PYL/RCAR–PP2C–SnRK2* core signaling module, thereby promoting the biosynthesis and accumulation of osmoprotectants, such as soluble sugars and proline (Yoshida et al., 2014; Umezawa et al., 2010). These compounds contribute to the maintenance of cellular osmotic balance, stabilization of membrane systems, and alleviation of dehydration-induced damage. In parallel, ABA-mediated signaling can enhance the activities of antioxidant enzymes, including superoxide dismutase, peroxidase, and catalase, thereby facilitating reactive oxygen species scavenging and reducing membrane lipid peroxidation and oxidative cellular damage (Gill and Tuteja, 2010; Mittler, 2002).

In the present study, transgenic tobacco plants overexpressing *AsGRF17* exhibited significantly higher soluble sugar content and increased SOD and POD activities than wild-type plants following treatment with 15% PEG6000 to simulate drought stress. Conversely, the levels of H_2_O_2_ and MDA were significantly lower in the *AsGRF17* overexpressing lines than in the wild type. These results indicate that *AsGRF17* overexpression may enhance drought tolerance by promoting osmotic adjustment, maintaining membrane stability, and strengthening the antioxidant enzyme system. Previous studies have shown that the miR396–GRF module is an important regulatory module involved in both plant development and stress adaptation (Casadevall et al., 2013; Liebsch and Palatnik, 2020). Under abiotic stress conditions, including drought, miR396 is often upregulated and suppresses the expression of GRF genes, thereby restricting cell proliferation and organ growth as part of the plant stress response (Casadevall et al., 2013; Bazin et al., 2013). However, the enhanced drought tolerance observed in *AsGRF17* overexpressing plants suggests that *AsGRF17* may function not only as a growth-promoting regulator but also as a component associated with stress-response networks, potentially including ABA-dependent signaling pathways. By maintaining relatively high metabolic activity, promoting the accumulation of osmoprotectants, and enhancing antioxidant enzyme activities, *AsGRF17* may improve plant adaptation to drought stress and partially compensate for the inhibitory effects of miR396-mediated *GRF* repression under adverse conditions. Further experimental validation, such as ABA-sensitivity assays, expression analysis of ABA-responsive genes, and verification of the miR396–AsGRF17 regulatory relationship, will be required to clarify the underlying molecular mechanism.

In this study, the drought-tolerance function of the oat *AsGRF17* gene was heterologously validated using a tobacco genetic transformation system. The results suggest that *AsGRF17* can enhance plant drought tolerance to some extent. However, because tobacco and oat differ in their biological characteristics and genetic backgrounds, the functional evidence obtained from the tobacco heterologous expression system may have limitations when extrapolated to oat. In addition, overexpression using a GFP-tagged expression vector may potentially affect the stability and/or functional activity of the target protein in transgenic lines. Therefore, future studies will focus on constructing an *AsGRF17* overexpression vector without any exogenous tag and further validating its function within the oat genetic background. In future studies, virus-induced gene silencing (VIGS)-based transient transformation assays will be performed to further investigate the function of *AsGRF17* in enhancing plant drought tolerance. Specifically, *AsGRF17* will be specifically silenced and subjected to drought stress treatments, followed by systematic evaluation of drought-related phenotypes, physiological parameters, and molecular responses, thereby providing further evidence for the biological role of *AsGRF17* in improving drought tolerance. These studies will enable a systematic evaluation of the role of *AsGRF17* in drought tolerance in oat lines, as well as comparative analyses with the phenotypic and physiological responses observed in transgenic tobacco. Overall, the present findings provide preliminary evidence that *AsGRF17* enhances plant drought tolerance and may serve as a valuable genetic resource for drought-resistance molecular breeding and genetic improvement in oat.

## 5 Conclusion

In summary, this study performed a genome-wide identification of 21 *AsGRFs* genes in oat and further classified them into five subfamilies. Members within the same subfamily exhibited highly conserved domain architectures and motif compositions. The cis-acting elements of *AsGRFs* are associated with responses to abiotic stress, hormones, and light, suggesting that *GRF* genes play important roles in oat adaptation to abiotic stresses and ABA signaling. Phenotypic analysis of *AsGRF17* demonstrated that it positively regulates plant drought tolerance. Specifically, *AsGRF17* overexpression increased soluble sugar content and SOD and POD activities, while decreasing MDA and H_2_O_2_ levels, thereby enhancing antioxidant capacity, alleviating membrane damage, and improving osmotic regulation. These findings highlight *AsGRF17* as a promising candidate for genetic improvement of drought tolerance in oat and provide a physiological framework for understanding its role under drought stress.

## Data Availability

The gene sequence is available in the NCBI, with GenBank accession No. PX841308. The data generated or analyzed during this study are included in this article and its **Supplementary Material**.
