# Supplementary material for: Genome-wide identification and characterization of the growth-regulating factor gene family in oat (Avena sativa L.) and functional analysis of AsGRF17 under drought stress
Source: Front Plant Sci. 2026 Aug 12;17:1885755. doi: 10.3389/fpls.2026.1885755 (PMC13506410; doi:10.3389/fpls.2026.1885755)
Supplement: Supplementary file 1 [file SupplementaryFile1.docx]

**Figure S1** Logos of 10 motifs in oat.

**Figure S2** Electrophoretic gel chart for identifying transgenic tobacco lines with Bar via PCR.
