# Supplementary material for: Genome-wide identification and characterization of the growth-regulating factor gene family in oat (Avena sativa L.) and functional analysis of AsGRF17 under drought stress
Source: Front Plant Sci. 2026 Aug 12;17:1885755. doi: 10.3389/fpls.2026.1885755 (PMC13506410; doi:10.3389/fpls.2026.1885755)
Supplement: Supplementary file 2 [file SupplementaryFile2.docx]

**Table S1** List of primers for PCR in this study.

| **Primer name** | **Sequence (5' to 3')** |
| --- | --- |
| AsGRF02-Q-F | GGAGGACAAGAAGGACACTAATC |
| AsGRF02-Q-R | GAATTCTTGGCCGGTTTCTTG |
| AsGRF04-Q-F | AGTCTCGCGTCTACCAGTAT |
| AsGRF04-Q-R | TTCAGAGGATGCACCAGTAAC |
| AsGRF06-Q-F | GAGCTTGTTGAGGATGGAGAA |
| AsGRF06-Q-R | GCTCAGAGACGATGAAAGGTAG |
| AsGRF09-Q-F | AGCTTGTTGATGACGGAGAG |
| AsGRF09-Q-R | AGAGACGACGACAGGTAGTT |
| AsGRF10-Q-F | CATCGATTCTCCATCGGATAGC |
| AsGRF10-Q-R | ACCTTGTAGGAGGCCAGTAT |
| AsGRF11-Q-F | TTCCAGAACCACTCCCTGTA |
| AsGRF11-Q-R | GTGCAGCTGAGATTGAGAAGA |
| AsGRF13-Q-F | ACAGGGACGTTGTACATCAC |
| AsGRF13-Q-R | CGCGTTGTTGTTGTTGCT |
| AsGRF17-Q-F | CGATCCTTGCTCACTGCAATA |
| AsGRF17-Q-R | CCCGTATTCCTTGCACTCATAG |
| AsGRF18-Q-F | GCAGAGATTCTTGCTGTCTAGG |
| AsGRF18-Q-R | GCCTTGGCGTTGAGATACTT |
| AsGRF19-Q-F | CTGCTTCCCACAAACCAAAC |
| AsGRF19-Q-R | GTTGCTGGAGAAGTGGAGATAG |
| AsGRF20-Q-F | ATGATGCTAAGAGGGAAAGCC |
| AsGRF20-Q-R | GGCGGCCATGTACTTGTAG |
| AsGRF21-Q-F | GGATAGCTCATGGCACACAA |
| AsGRF21-Q-R | CGACGGTTCAAACTGGGAATA |
| AsUBC-Q-F | ACAGCGTTCCGAGAGTTGTT |
| AsUBC-Q-R | AAGAGTCAGCCCGAATGCAG |
| AsGRF17-F | ATGGACCTGGGCGGGG |
| AsGRF17-R | TCACAGCAGGCGGATGCT |
| PC1301S-AsGRF17-F | atgatgatgataaaggtaccATGGACCTGGGCGGGG |
| PC1301S-AsGRF17-R | ctagaggatccccgggtaccCAGCAGGCGGATGCT |
| Ntactin-F | TTTGAGACTTTCAATGTGCCCGCC |
| Ntactin-R | TAGCATGTGGGAGTGCATAACCCT |
| barGRF-F | TCATGCCAGTTCCCGTGCTT |
| barGRF-R | GGTCTGCACCATCGTCAACCA |

**Table S2** Basic information on the 21 AsGRFs identified in this study.

| Name | Sequence ID | Number of  Amino Acid | MW（KDa） | PI | Instability Index | Aliphatic  Index | Grand Average  of Hydropathicity | Subcellular location |
| --- | --- | --- | --- | --- | --- | --- | --- | --- |
| AsGRF01 | AVESA.00001b.r3.2Ag0000290.2 | 321 | 34.17 | 4.97 | 67.07 | 59.03 | -0.614 | Nuclear |
| AsGRF02 | AVESA.00001b.r3.2Ag0000290.1 | 265 | 28.10 | 4.84 | 58.41 | 60.45 | -0.674 | Nuclear |
| AsGRF03 | AVESA.00001b.r3.2Cg0001191.1 | 168 | 18.38 | 9.46 | 54.02 | 63.33 | -0.632 | Nuclear |
| AsGRF04 | AVESA.00001b.r3.2Dg0002858.1 | 264 | 29.32 | 9.16 | 48.89 | 89.77 | -0.133 | Nuclear |
| AsGRF05 | AVESA.00001b.r3.2Dg0002858.2 | 328 | 34.97 | 4.85 | 68.02 | 61.31 | -0.591 | Nuclear |
| AsGRF06 | AVESA.00001b.r3.4Ag0001381.3 | 561 | 61.07 | 6.5 | 54.37 | 75.33 | -0.258 | Nuclear |
| AsGRF07 | AVESA.00001b.r3.4Ag0001381.1 | 585 | 62.34 | 6.19 | 53.45 | 70.48 | -0.378 | Nuclear |
| AsGRF08 | AVESA.00001b.r3.4Ag0001381.2 | 593 | 63.34 | 6.29 | 53.26 | 70.19 | -0.364 | Nuclear |
| AsGRF09 | AVESA.00001b.r3.4Dg0001724.1 | 587 | 62.53 | 6.19 | 53.21 | 69.4 | -0.391 | Nuclear |
| AsGRF10 | AVESA.00001b.r3.5Cg0003282.1 | 350 | 38.27 | 8.79 | 62.27 | 51.4 | -0.697 | Nuclear |
| AsGRF11 | AVESA.00001b.r3.6Ag0001106.1 | 408 | 43.58 | 8.64 | 61.52 | 57.3 | -0.558 | Nuclear |
| AsGRF12 | AVESA.00001b.r3.6Ag0001786.1 | 424 | 45.85 | 7.79 | 49.1 | 49.81 | -0.736 | Nuclear |
| AsGRF13 | AVESA.00001b.r3.6Dg0001477.1 | 420 | 45.62 | 7.79 | 48.61 | 49.36 | -0.756 | Nuclear |
| AsGRF14 | AVESA.00001b.r3.7Ag0000611.1 | 425 | 46.40 | 9.24 | 70.78 | 54.02 | -0.69 | Nuclear |
| AsGRF15 | AVESA.00001b.r3.7Ag0000611.2 | 292 | 32.12 | 9.55 | 74.35 | 55.58 | -0.68 | Nuclear |
| AsGRF16 | AVESA.00001b.r3.7Cg0000897.4 | 455 | 49.73 | 6.5 | 57.79 | 69.93 | -0.531 | Nuclear |
| AsGRF17 | AVESA.00001b.r3.7Cg0000897.1 | 588 | 62.77 | 6.31 | 54.36 | 70.12 | -0.395 | Nuclear |
| AsGRF18 | AVESA.00001b.r3.7Cg0001177.2 | 346 | 39.39 | 9.13 | 57.75 | 52.2 | -1.073 | Nuclear |
| AsGRF19 | AVESA.00001b.r3.7Cg0001177.1 | 463 | 51.75 | 9.14 | 59.5 | 55.75 | -0.847 | Nuclear |
| AsGRF20 | AVESA.00001b.r3.7Dg0003531.1 | 349 | 38.11 | 8.95 | 62.98 | 50.69 | -0.696 | Nuclear |
| AsGRF21 | AVESA.00001b.r3.7Dg0000064.1 | 325 | 35.66 | 8.6 | 68.64 | 46.98 | -0.86 | Nuclear |

**Table S3** List of 10 motifs with basic information.

| **Motif** | **E-value** | **Sites** | **Width** |
| --- | --- | --- | --- |
| 1 | 1.3e-818 | 21 | 50 |
| 2 | 6.3e-566 | 21 | 50 |
| 3 | 2.2e-161 | 8 | 41 |
| 4 | 1.9e-136 | 6 | 36 |
| 5 | 1.2e-182 | 6 | 50 |
| 6 | 1.2e-157 | 8 | 50 |
| 7 | 1.8e-138 | 13 | 41 |
| 8 | 8.6e-121 | 6 | 42 |
| 9 | 2.9e-105 | 6 | 29 |
| 10 | 3.4e-090 | 9 | 31 |

**Table S4** The Ka/Ks ratios of AsGRF collinear gene pairs in oat.

| **Sequence 1** | | **Sequence 2** | | **Ka** | **Ks** | **Ka/Ks** |
| --- | --- | --- | --- | --- | --- | --- |
| **Name** | **ID** | **Name** | **ID** |  |  |  |
| AsGRF01 | AVESA.00001b.r3.2Ag0000290.2 | AsGRF13 | AVESA.00001b.r3.2Cg0001191.1 | 0.018568588 | 0.059521306 | 0.311965397 |
| AsGRF01 | AVESA.00001b.r3.2Ag0000290.2 | AsGRF05 | AVESA.00001b.r3.2Dg0002858.2 | 0.001373941 | 0.012903544 | 0.106477825 |
| AsGRF03 | AVESA.00001b.r3.2Cg0001191.1 | AsGRF05 | AVESA.00001b.r3.2Dg0002858.2 | 0.018568588 | 0.050723551 | 0.366074288 |
| AsGRF06 | AVESA.00001b.r3.4Ag0001381.2 | AsGRF09 | AVESA.00001b.r3.4Dg0001724.1 | 0.004542314 | 0.073213948 | 0.062041649 |
| AsGRF06 | AVESA.00001b.r3.4Ag0001381.2 | AsGRF17 | AVESA.00001b.r3.7Cg0000897.1 | 0.019556323 | 0.144085596 | 0.135727118 |
| AsGRF09 | AVESA.00001b.r3.4Dg0001724.1 | AsGRF17 | AVESA.00001b.r3.7Cg0000897.1 | 0.017988998 | 0.130515864 | 0.137829972 |
| AsGRF10 | AVESA.00001b.r3.5Cg0003282.1 | AsGRF14 | AVESA.00001b.r3.7Ag0000611.1 | 0.010265344 | 0.13188049 | 0.077838232 |
| AsGRF10 | AVESA.00001b.r3.5Cg0003282.1 | AsGRF21 | AVESA.00001b.r3.7Dg0003531.1 | 0.01672095 | 0.108295879 | 0.154400611 |
| AsGRF12 | AVESA.00001b.r3.6Ag0001786.1 | AsGRF13 | AVESA.00001b.r3.6Dg0001477.1 | 0.007404203 | 0.071018944 | 0.104256731 |
| AsGRF12 | AVESA.00001b.r3.6Ag0001786.1 | AsGRF20 | AVESA.00001b.r3.7Dg0000064.1 | 0.271728774 | 0.392788634 | 0.691793882 |
| AsGRF13 | AVESA.00001b.r3.6Dg0001477.1 | AsGRF20 | AVESA.00001b.r3.7Dg0000064.1 | 0.275356214 | 0.393194987 | 0.700304487 |
| AsGRF14 | AVESA.00001b.r3.7Ag0000611.1 | AsGRF21 | AVESA.00001b.r3.7Dg0003531.1 | 0.008984065 | 0.077779245 | 0.115507223 |
